# Quantitative analysis of opioids and cannabinoids in wastewater samples

**DOI:** 10.1080/20961790.2016.1270812

**Published:** 2017-01-30

**Authors:** Alethea Jacox, Jillian Wetzel, Shu-Yuan Cheng, Marta Concheiro

**Affiliations:** Department of Sciences, John Jay College of Criminal Justice, City University of New York, New York, NY, USA

**Keywords:** Forensic science, forensic toxicology, chromatography liquid, tandem mass spectrometry, solid phase extraction, wastewater, opioid, cannabis

## Abstract

Wastewater-based epidemiology is an innovative approach that uses the analysis of human excretion products in wastewater to obtain information about exposure to drugs in defined population groups. We developed and validated an analytical method for the simultaneous determination of opioids (morphine, oxycodone, hydrocodone, oxymorphone and hydromorphone), and cannabinoids (Δ^9^-tetrahydrocannabinol, 11-*nor*-9-carboxy-tetrahydrocannabinol (THCCOOH) and THCCOOH-glucuronide) in raw-influent wastewater samples by ultra-high performance liquid chromatography-tandem mass spectrometry. Method validation included linearity (5–1 000 ng/L for opioids, 10–1 000 ng/L for cannabinoids), imprecision (<21.2%), accuracy (83%–131%), matrix effect (from –35.1% to –14.7%) and extraction efficiency (25%–84%), limit of detection (1–5 ng/L) and quantification (5–10 ng/L) and auto-sampler stability (no loss detected). River and wastewater samples were collected in triplicate from different locations in New York City and stored at −20 °C until analysis. Water from sewage overflow location tested positive for morphine (10.7 ng/L), oxycodone (4.2–23.5 ng/L), oxymorphone (4.8 ng/L) and hydromorphone (4.2 ng/L). Raw influent wastewater samples tested positive for morphine (133.0–258.3 ng/L), oxycodone (31.1–63.6 ng/L), oxymorphone (16.0–56.8 ng/L), hydromorphone (6.8–18.0 ng/L), hydrocodone (4.0–12.8 ng/L) and THCCOOH (168.2–772.0 ng/L). This method is sensitive and specific for opioids and marijuana determination in wastewater samples.

## Introduction

Wastewater analysis is the method of choice for determining what drug(s) are being used in the geographical areas that wastewater treatment plants service [1]. By observing human biomarkers in sewage water, analysts can monitor the consumption of various drugs. These findings can then be compared to, and even supplement, traditional anonymous surveys. Wastewater epidemiology/toxicology is inexpensive, provides virtually real-time data, and is reliable for assessing the extent of drug use in a geographical region of interest. Its ability to rapidly determine drug use trends in an area can help with the development of targeted public health programs and policy initiatives in these specific communities. However, some disadvantages of wastewater analysis include uncertainties because of population flow variations (e.g. with visitors and tourists), sewage flow changes, rainfall, and varying inter-individual drug excretion rates [2,3]. Whereas wastewater analysis is a rapidly growing field in Europe [4], data for the evaluation of raw influent wastewater in the United States (USA) are scarce [2,5]. This type of study has never been performed in New York City (NYC), which is the largest city in the USA.

Prescription opioids are used to treat chronic pain, and their use has increased dramatically in recent years. This has been strongly associated with increasing rates of nonmedical use of prescription opioids in the USA [6]. This situation has led to opioids being the most abused class of prescription drugs [7]. According to statistics from the New York City Health Department, 59 opioid-related deaths occurred in 2000, and this increased to 220 opioid-related deaths in 2013 [8]. Between 2005 and 2014, the rate of deaths because of prescription opioid increased 250% (rate of increase per 100 000 general population). In 2005, prescription opioids contributed to 29% of the drug overdoses in New York, and this figure rose to 43% by 2014 [9]. According to the 2014 National Survey on Drug Use and Health [10], 4.3 million people aged 12 or older have reported current nonmedical use of prescription pain relievers.

In the USA, marijuana is the most commonly used illicit drug, with 22.2 million marijuana users aged 12 or older that have used the drug in the past month (past-month users) [10]. This is followed by stimulants (1.6 million past-month users), cocaine (1.5 million past-month users) and heroin (400 000 past-month users). Based on National Statistics, 44% of adolescents 12 years and older have used marijuana in their lifetime, which is about the same percentage as individuals aged 26 and older. Individuals aged 18 to 25 years old have the highest percentage of marijuana users (52%) [11]. On July 7 2014, New York became the 23rd state to legalize medical marijuana [12], allowing medical facilities in eight cities to prescribe capsules, liquids, oils, or vaporizable forms of cannabis. The effect of marijuana legalization on prevalence of use is still unknown.

Several authors have published methods for the determination of licit and illicit drugs in wastewater [13–28]. However, prescription opioid data are scarce, and wastewater samples have never been analysed for the major cannabis metabolite in human urine, 11-*nor*-9-carboxy-tetrahydrocannabinol-glucuronide (THCCOOH-glucuronide). The objective of this study was to develop and validate an analytical method for the detection of morphine, common prescription opioids (oxycodone, oxymorphone, hydrocodone and hydromorphone) and cannabinoids in wastewater samples. Then, for proof of concept, this method was applied to river water and raw influent wastewater samples collected from different locations within NYC.

## Materials and methods

### Reagents and materials

Morphine, oxycodone, hydrocodone, oxymorphone, hydromorphone, Δ^9^-tetrahydrocannabinol (THC) and its metabolites 11-*nor*-9-carboxy-tetrahydrocannabinol (THCCOOH) and THCCOOH-glucuronide were purchased from Cerilliant (Round Rock, TX, USA). The deuterated analogs THC-d_3_, THCCOOH-d_3_, THCCOOH-glucuronide-d_3_, morphine-d_3_, oxycodone-d_6_, hydrocodone-d_6_, oxymorphone-d_3_ and hydromorphone-d_6_ were also purchased from Cerilliant. Strata XC 33 µm polymeric strong cation exchange cartridges of 3 mL/60 mg for calibrators and 6 mL/200 mg for quality control (QC) and authentic wastewater samples were purchased from Phenomenex (Torrance, CA, USA). Ultra-high performance liquid chromatography (UHPLC) grade methanol, dichloromethane and ammonium hydroxide were purchased from Pharmco-Aaper (Brookfield, CT). Isopropanol, liquid chromatography mass spectrometry grade acetonitrile, and Whatman glass microfiber filters (outside diameter 4.7 cm, particle retention 1.6 μm, and thickness 0.26 mm) were from Thermo Fisher Scientific (Waltham, MA, USA).

### Instrumentation

The chromatographic separations were carried out on an UHPLC–tandem mass spectrometry (MS/MS) instrument from Shimadzu (Kyoto, Japan). The Nexera UHPLC system consisted of a binary LC-20ADXR high-performance liquid chromatography pump, Nexera LC-30AD micro mixer, online degassing unit (DGU-20A3R) and cooled autosampler (SIL-20SCHT UFLC). The chromatographic column was a Kinetex C18 (2.1 mm × 100 mm, 1.7 µm particle size, 100 Å pore size) and the guard column was a SecurityGuard ULTRA Cartridges C18(2.1 mm × 2 mm, Phenomenex). Mobile phase A was 0.1% formic acid in water and mobile phase B was 0.1% formic acid in acetonitrile. The following gradient program was used for elution of cannabinoids: held at 40% B for 4 min, increased to 95% B and held for 1 min, decreased to 40% B in 0.5 min and held at 40% B for 1.5 min. The total run time was 7 min and the mobile phase flow rate was 0.5 mL/min. The following gradient program was used for elution of opioids: held at 2% B for 1 min, increased to 30% B in 3 min, increased to 95% B in 2 min and held for 1 min, decreased to 2% B in 0.5 min and held for 2.5 min. The total run time was 10 min and the mobile phase flow rate was 0.3 mL/min. The column oven was operated at 40 °C. The injection volume was 50 µL for each set of compounds.

The mass spectrometer was a triple quadrupole LC-MS 8030 from Shimadzu equipped with a dual ionization source (atmospheric pressure chemical and electrospray ionization). The nebulizing gas flow was set to 2 L/min, the desolvation line was at 250 °C, the heating block was at 400 °C and the drying gas flow was at 15 L/min. The dual ionization source corona needle voltage and interface voltage were both set to 4.5 kV. Two multiple reaction monitoring (MRM) transitions were monitored for each compound (Table 1), with one used as a quantifier and the other as a qualifier.

**Table 1.** Multiple reaction monitoring transitions, retention time (RT) and precursor ion for each analyte of interest.

Compound	RT (min)	Precursor ion (*m/z*)	Quantifier product ion (*m/z*)	Collision energy^1)^ (eV)	Qualifier product ion (*m/z*)	Collision energy^2)^ (eV)
Morphine	3.14	286	165	−40	181	−33
Morphine-d_3_	3.13	289	164	−44	153	−43
Hydromorphone	3.44	286	184	−33	157	−43
Hydromorphone-d_6_	3.42	292	185	−33	157	−49
Oxymorphone	3.29	302	226	−32	242	−28
Oxymorphone-d_3_	3.29	304	201	−45	230	−31
Oxycodone	3.98	316	256	−26	212	−46
Oxycodone-d_6_	3.97	322	247	−31	262	−29
Hydrocodone	4.10	300	199	−31	170	−40
Hydrocodone-d_6_	4.08	306	202	−36	174	−44
THC	4.01	315	193	−23	122	−38
THC-d_3_	4.00	318	195	−27	122	−39
THCCOOH	2.87	345	299	−21	192	−28
THCCOOH-d_3_	2.86	348	330	−17	302	−23
THCCOOH-glucuronide	1.98	521	345	−15	326	−18
THCCOOH-glucunoride-d_3_	1.94	524	348	−15	330	−21

^1)^Collision energy for Quantifier production.

^2)^Collision energy for Qualifier production.

### Sample preparation

An aliquot (100 mL) of each wastewater sample was measured using a graduated cylinder and placed in a beaker, spiked with 50 µL of internal standard mixture (0.1 μg/mL), and filtered through a glass microfiber filter. Then, 0.5 mL of HCl was added immediately to acidify the solution to maximize retention onto mixed-mode cartridges.

### Solid phase extraction

Strata XC 6 mL/200 mg cartridges were conditioned with 6 mL of methanol and then 6 mL of ultra-high purity (UHP) water, and 6 mL of 0.1% HCl. Then the 100 mL of acidified wastewater was manually loaded 6 mL at a time (17 times) onto a cartridge with a small vacuum (<34 473 Pa). The cartridges were washed with 4 mL of UHP water and 4 mL of 0.1% HCl, and then dried under vacuum for 15 min. Finally, 8 mL of elution solvent (*V*_dichloromethane_:*V*_isopropanol_:*V*_ammonium hydroxide_ = 78:20:2) was added. A vacuum was applied to retrieve all the solvent, and the eluate was split in half. The opioid samples were labelled set 1, and the cannabinoid samples were labelled set 2. Each set was evaporated to dryness under a steady stream of N_2_ in a Biotage TurboVap (Uppsala, Sweden) at 40 °C. The opioid samples (set 1) were reconstituted in 200 µL of UHP water, and the cannabinoid samples (set 2) were reconstituted in 200 µL of a mixture (*V*_A_:*V*_B_ = 60:40) of mobile phases A (0.1% formic acid in water) and B (0.1% formic acid in acetonitrile).

### Calibrators, quality controls and internal standards

An internal standard working solution was prepared by diluting each ampoule with pure methanol and combining all analogues to a final concentration of 0.1 µg/mL in methanol. Standard working solutions were prepared in pure methanol to a stock concentration of 1 µg/mL, and then serially diluted to concentrations of 0.1 and 0.01 µg/mL in pure methanol. Calibration working solution mixtures, containing all compounds of interest, were also prepared in methanol to concentrations of 0.001, 0.01, 0.1, and 1 µg/mL. These solutions were used to prepare standard curve solutions at concentrations of 0.5, 1, 3, 5, 10, 50, 100, 500 and 1 000 ng/L.

To reduce the cost and time of this process, calibrators were prepared in 3 mL of UHP water spiked with the corresponding calibration working solution to match the amounts of 5 to 1 000 ng/L in 100 mL of sample. For the calibration curve, clean test tubes were prepared containing 3 mL of UHP water, 50 µL of internal standard mixture (0.1 μg/mL), and the following volumes of the respective calibrator working solution: 50 and 100 µL of the 0.01 µg/mL solution for 5 and 10 ng/L calibrators, 50 and 100 µL of the 0.1 µg/mL solution for 50 and 100 ng/L calibrators, and 50 and 100 µL of the 1 µg/mL solution for 500 and 1 000 ng/L calibrators. Lastly, 15 µL of HCl (0.5%) was added before vortex mixing and solid phase extraction (SPE). Strata XC 3 mL/60 mg cartridges were conditioned with 3 mL of methanol, followed by 3 mL of UHP water and 3 mL of 0.1% HCl. The acidified calibrator was loaded onto the mixed-mode cartridge. Cartridges were washed with 2 mL of UHP water and 2 mL of 0.1% HCl, and then dried under vacuum for 15 min. Sample elution was performed with 4 mL of a dichloromethane/isopropanol/ammonium hydroxide mixture (*V*_dichloromethane_:*V*_isopropanol_:*V*_ammonium hydroxide_ = 78:20:2). The eluate was split in half. The samples were labelled as set 1 for opioids and set 2 for cannabinoids. Each set was evaporated to dryness under a steady stream of N_2_ in a Biotage TurboVap at 40 °C. Opioid samples (set 1) were reconstituted in 200 µL of UHP water, and cannabinoids samples (set 2) were reconstituted in 200 µL of a mixture (*V*_A_:*V*_B_ = 60:40) of mobile phases A (0.1% formic acid in water) and B (0.1% formic acid in acetonitrile).

QC samples were prepared at 10 and 100 ng/L by spiking 100 mL of UHP water with the required amount of the working solution and 50 µL of the internal standard mixture. These samples were then filtered, and 0.5 mL of HCl (0.5% HCl) was added before SPE.

### Sample collection

For proof of concept, 33 samples were collected from river water (22 samples), sewage overflow (6 samples) and raw influent from wastewater treatment plants (5 samples) in NYC. River samples were collected from the Hudson and East Rivers in the Bronx, Manhattan, Queens and Roosevelt Island. Sewage overflow samples were collected from Newtown Creek (Brooklyn), and wastewater samples were collected from the Tallman and Jamaica wastewater treatment plants in Queens. Samples were collected for 1–3 days before and after national holidays (Independence Day, July 4 2015; Labor Day, September 7 2015; New Year's Day, January 1 2016) and on March 25th and 30th 2016. The samples were collected at one time on each of these days (between 7 and 11 am) in 200 mL Nalgene™ Certified Wide-Mouth Amber high-density polyurethane bottles (Thermo Fisher Scientific). To prevent degradation of the target drugs, the samples were stored in a freezer at –20 °C until required for analysis.

#### Validation parameters

The method was validated using various procedures outlined by the Scientific Working Group for Forensic Toxicology guidelines [29] for the linearity, limit of detection (LOD), limit of quantification (LOQ), interferences (specificity), autosampler stability, imprecision, accuracy, carryover, extraction efficiency, process efficiency and matrix effect.

Linearity was determined over five different days by least-squares regression and different weighting factors (none, 1/*x* and 1/*x*^2^) were evaluated. The linearity was acceptable if the coefficient of determination (*R*^2^) was ≥ 0.99 and the residuals were within ±20%. The LOD and the LOQ were evaluated with decreasing analyte concentrations in spiked samples from three different sources. The LOD was the lowest concentration with acceptable chromatographic parameters, a signal-to-noise ratio > 3, the presence of all product ions, the correct ion ratio (within ±20% from the average of the calibrators) and a suitable retention time (within ±0.2 min of the retention time of the calibrators). The LOQ satisfied the LOD criteria and was quantified within ±20% imprecision and 80%–120% accuracy.

Interferences from matrix components were evaluated by analysing river (*n* = 22) and wastewater (*n* = 4) samples negative for the compounds of interest, after spiking with the internal standard solution. Interferences were considered insignificant if the analytes of interest were not detected in these samples. Method specificity was demonstrated by analysing high concentrations (1 000 ng/L) of potentially interfering drugs. The following compounds and their metabolites were examined: opioids (morphine-3-glucuronide, morphine-6-glucuronide, hydromorphone-3-glucuronide, oxymorphone-3-glucuronide, oxymorphone-6-glucuronide and 6-acetylmorphine), cannabinoids (11-hydroxy-THC, cannabinol, and cannabidiol) and common drugs of abuse (cocaine, benzoylecgonine, amphetamine, methamphetamine, 3,4-methylenedioxyamphetamine, 3,4-methylenedioxymethamphetamine and methadone). Sufficient specificity was achieved if the analytes of interest were below the LOD.

To determine carryover, blank samples spiked with the internal standard (negative calibrator) were injected immediately after samples spiked at 2 000 ng/L (twice the highest calibrator concentration). The carryover was considered negligible if the measured concentration was less than the LOD. Before SPE, the 2 000 ng/L samples were prepared using 3 mL of UHP water and spiking it with 50 µL of internal standard, 200 µL of the 1 µg/mL calibrator solution and 15 µL of HCl.

Inter- and intra-day QC samples at 10 ng/L and 100 ng/L were prepared in 100 mL of UHP water spiked with 100 µL of the 0.01 µg/mL solution (for 10 ng/L concentration) or the 0.1 µg/mL solution (for 100 ng/L concentration). The imprecision and accuracy were determined at these two concentrations with four repeat analyses on one day (intra-day *n* = 4) and over five days (inter-day *n* = 5). The imprecision was determined using the coefficient of variation of the measured values and expected to be less than 20%. The intra- and inter-day imprecision were calculated as the standard deviation of the QC concentrations × 100/mean QC concentrations. The accuracy was calculated as a percentage of the target concentration, and was required to be within 80%–120%. The intra- and inter-day accuracy was calculated as the mean QC concentrations × 100/QC target concentration.

Autosampler stability was evaluated by reinjecting four QC samples after 24 h in the autosampler at 10 °C. The QC samples were prepared at 10 ng/L using 100 mL of UHP water and 100 µL of 0.01 µg/mL calibrator working solution. The concentrations within ±20% of the initial concentration were considered acceptable.

To evaluate the matrix effect, extraction efficiency and process efficiency, three sets of samples were prepared in duplicate at the same concentration (10 ng/L). Set 1 contained neat samples prepared by adding 2 mL of elution solvent, 50 µL of internal standard, and 100 µL of the 0.01 µg/mL solution to a clean test tube. This sample was then split equally, evaporated to dryness and reconstituted in the appropriate opioid or cannabis mobile phase for LC-MS/MS separation and analysis. Set 2 contained QC samples spiked at 10 ng/L with the internal standard and submitted to the same sample preparation and extraction steps as above. Set 3 contained QC samples spiked at 10 ng/L and with the internal standard post-extraction. The samples were from four different sources, including samples prepared with UHP water (one set) and using authentic wastewater samples that tested negative for the target drugs (three sets). The peak areas for Set 1 and 3 were compared to determine if there were any matrix effects. The peak areas for the Set 2 and 3 samples were compared to assess the extraction efficiency, and those for Set 1 and Set 2 were used to assess the process efficiency.

### Identification criteria

The identification criteria included a retention time within ±0.2 min of the calibrator retention time, the presence of two product ions (quantitative and qualitative) and an ion ratio within ±20% from the average of the calibrators.

## Results

### Method validation

The LOQ and LOD for all opioids were 5 and 1 ng/L, respectively, and the linear range was 5–1 000 ng/L. For the cannabinoids, the LOQ, LOD and linear range were 10, 5 and 10–1 000 ng/L, respectively. Acceptable linearity for opioids and cannabinoids (*R*^2^ ≥ 0.99 and residuals within ±20%) were achieved with 1/*x*^2^ weighting. No endogenous or exogenous interferences were detected.

For opioids, the intra- and inter-day imprecision were 3.3% to 14.1%, respectively, and the accuracy was 93.3%–131.0%. For cannabinoids, the intra and inter-day imprecision were 4.1% to 21.2%, respectively, and the accuracy was 83.0% to 119.3%. For opioids, the extraction efficiency range was 75.0%–84.0%, and the process efficiency range from 63.1% to 73.3%. For cannabinoids, the extraction efficiency range was 25.4%–66.5% and the process efficiency range was 22.7%–62.7%. For opioids, the matrix effects range was –35.1% to –7.6% (ion suppression), with a coefficient of variation of ≤ 28.3% (*n* = 4). For cannabinoids, the matrix effects range was –14.7% to –5.8%, with a coefficient of variation of ≤ 13.9% (*n* = 4). These results are summarized in Tables 2 and 3.

**Table 2.** Inter- and intra-day imprecision and accuracy for quality controls at 10 and 100 ng/L.

	Imprecision (%)				Accuracy (%)			
	Inter-day (*n* = 5)		Intra-day (*n* = 4)		Inter-day (*n* = 5)		Intra-day (*n* = 4)	
Compound	10 (ng/L)	100 (ng/L)	10 (ng/L)	100 (ng/L)	10 (ng/L)	100 (ng/L)	10 (ng/L)	100 (ng/L)
Morphine	11.4	6.2	5.6	3.4	93.3	94.7	116.0	110.9
Oxymorphone	10.8	3.9	4.9	3.3	106.5	106.6	126.5	109.1
Hydromorphone	14.1	3.3	12.9	4.4	98.3	103.3	119.5	121.1
Oxycodone	8.9	7.6	10.9	5.0	98.3	101.1	110.0	102.0
Hydrocodone	10.0	8.8	6.1	4.8	107.5	103.7	131.0	127.2
THC	9.7	9.0	21.2	7.9	102.8	98.8	110.3	106.6
THCCOOH	8.0	6.1	5.0	7.3	97.3	105.6	119.3	103.4
THCCOOH-glucuronide	6.0	10.2	4.1	5.5	95.0	98.9	83.0	102.3

**Table 3.** Extraction efficiency, process efficiency, matrix effect and coefficient of variation for each analyte at 10 ng/L.

Compound	Extraction efficiency (%)	Process efficiency (%)	Matrix effect (%)
Morphine	79.0	73.3	−7.6
Oxymorphone	84.0	63.1	−24.5
Hydromorphone	75.0	48.8	−35.1
Oxycodone	82.0	63.5	−23.1
Hydrocodone	84.0	72.6	−13.8
THC	25.4	22.7	−10.6
THCCOOH	66.5	62.7	−5.8
THCCOOH-glucuronide	53.1	45.3	−14.7

Carryover was assessed by injecting a blank after injection of a sample prepared at 2 000 ng/L (twice the concentration of our highest calibrator). The results for all target compounds for the blank were below LOD, which means that all extracted compounds are eluted before injection of the next sample. Autosampler stability was assessed by injecting the same both fresh and after 24 h in the autosampler at 10 °C. The mean concentrations from these injections were compared to determine the percentage difference. The concentrations of all target compounds were within the accepted 20%, except for oxymorphone (–21.3%).

### Application to authentic samples

**Figure 1.**
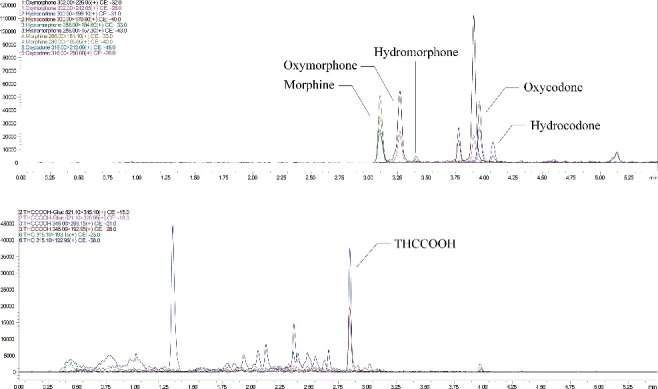
Multiple reaction monitoring chromatograms of an authentic wastewater sample from Tallman Island Wastewater Treatment Plant (Queens, NYC) showing positive results for THCCOOH (184.1 ng/L), morphine (181.9 ng/L), hydromorphone (10.0 ng/L), oxymorphone (56.8 ng/L), oxycodone (63.3 ng/L) and hydrocodone (10.3 ng/L).

Samples from the East and Hudson rivers tested negative for morphine, prescription opioids and cannabis. Samples from sewage overflows (Newtown Creek, Brooklyn) tested positive for morphine (10.7 ng/L), oxycodone (4.2–23.5 ng/L), oxymorphone (4.8 ng/L) and hydromorphone (4.2 ng/L). Raw influent wastewater samples from the Tallman and Jamaica plants in Queens tested positive for morphine (133.0–258.3 ng/L), oxycodone (31.1–63.6 ng/L), oxymorphone (16.0–56.8 ng/L), hydromorphone (6.8–18.0 ng/L), hydrocodone (4.0–12.8 ng/L) and THCCOOH (168.2–772.0 ng/L) (Table 4). Figure 1 shows a chromatogram of an authentic wastewater sample that tested positive for opioids and cannabinoids.

**Table 4.** Results from raw input wastewater plants (Tallman and Jamaica, New York City, NY) collected at one time point 1–3 days before and after national holidays (Independence Day, July 4 2015; Labor Day, September 7 2015; New Year's Day, January 1 2016) and in March 2016.

Analyte	Concentration range (ng/L)	*N* cases
Morphine	133.0 – 258.3	5
Hydrocodone	4.0 – 12.8	4
Oxycodone	31.1 – 63.6	5
Oxymorphone	16.0 – 56.8	5
Hydromorphone	6.8 – 18.0	5
THCCOOH	168.2 – 772.0	5

## Discussion

We developed and validated a method for the simultaneous analysis of morphine, oxymorphone, oxycodone, hydromorphone, hydrocodone, THC and its metabolites THCCOOH and THCOOH-glucuronide in wastewater samples. Numerous methods for the determination of licit and illicit drugs in wastewater samples have been published [13–28]. These analytical methods allow for the determination of opiates and prescription opioids [14,18,25,27,28], cannabis [16,19] or both classes of compounds, opiates and cannabis [13,15,18–20,21,22,24,26]. In the case of opiates, most of the methods can only detect morphine [13–15,17,18,20,21–26,28], although some methods are suitable for prescription opioids such as oxycodone, oxymorphone, hydrocodone and hydromorphone [14,18,19,25–28]. With regard to cannabis, most methods have been developed for THCCOOH [15,16,19,22,24] or for THC or THC and THCCOOH [13,17,20,21,23,26]. There is no data available for THCCOOH-glucuronide in wastewater samples, even though this compound is the predominant THC metabolite in human urine [30]. This may be because glucuronides are normally hydrolysed in wastewater [31], resulting in higher concentrations of the free compound. However, recent publications have reported high concentrations of glucuronides in wastewater samples [32,33]. These results highlight the need for a method for detection of glucuronides in wastewater.

Currently, the method most commonly used for wastewater analysis is LC-MS/MS. However, gas chromatography-mass spectrometry has been used too [34]. In the present method, all compounds were ionized in positive mode (atmospheric pressure chemical/electrochemical ionization dual source), despite other authors finding better sensitivity for cannabinoids in negative ionization mode (electrochemical ionization) [13,15,21,24]. The sensitivity of our method (LOD 1–5 ng/L and LOQ 5–10 ng/L in 100 mL of wastewater) was within the range of methods in previous publications [19,20,22,25]. Earlier studies have reported LOQs for the compounds of interest as low as 0.48 ng/L [35] and as high as 100 ng/L [22] for wastewater volumes between 15 mL [28] and 250 mL [17]. Sample preparation usually involves filtration and SPE with a reversed-phase (Oasis HLB, Waters Corp., Milford, MA, USA) or cation exchange (Oasis MCX, Waters Corp.) cartridges.

During method validation, matrix effect experiments were carried out using four different matrices instead of six [29]. This limitation was because it was difficult to obtain wastewater samples that were negative for the compounds of interest. Another limitation of the present method was the intra-day accuracy above the established criteria for three opioids. Although oxymorphone's low QC, hydromorphone's high QC and hydrocodone's low and high QC gave an intra-day accuracy >120% (121.1% to 131%), the rest of the validation parameters were within the established range [29].

As a proof of concept, we were able to detect THCCOOH, morphine and prescription opiates in water samples from sewage overflow locations and wastewater treatment plants. The concentrations of morphine (10.7–258.3 ng/L), oxycodone (4.2–63.6 ng/L), oxymorphone (4.8–56.8 ng/L), hydromorphone (4.2–18.0 ng/L), hydrocodone (4.0–12.8 ng/L) and THCCOOH (168.2–772.0 ng/L) were similar to those found in previous studies [18,19,28,29,33]. THC and THCCOOH-glucuronide were not detected in any of the analysed samples. Previously, THC was detected in wastewater samples [17], but there are no reports of the detection of THCCOOH-glucuronide. Continued research is required to investigate the importance of monitoring THCCOOH-glucuronide's in these types of samples, and to back calculate the drug exposure in communities based on wastewater drug concentrations.

## Conclusion

We developed and validated an analytical method for the simultaneous determination of morphine, common prescription opioids (oxycodone, oxymorphone, hydrocodone and hydromorphone), and cannabis and its metabolites in wastewater samples. This technique is sensitive (LOD 1–5 ng/L and LOQ 5–10 ng/L in 100 mL of sample) and specific. This is the first report of testing for THCCOOH-glucuronide in wastewater samples. As a proof of concept, we were able to detect THCCOOH, morphine, and prescription opioids in samples from sewage overflow locations and wastewater plants throughout NYC.
